# Deciphering the regulatory mechanisms of honeysuckle (*Lonicera japonica*) on lipopolysaccharide-induced inflammatory responses in loach (*Misgurnus anguillicaudatus*) through small RNA sequencing

**DOI:** 10.3389/fimmu.2025.1683771

**Published:** 2025-09-23

**Authors:** Ruike Fan, Zihan Sun, Lishang Dai, Xiajuan Jiang

**Affiliations:** ^1^ Department of Pharmacy, The First Affiliated Hospital of Wenzhou Medical University, Wenzhou, China; ^2^ School of Traditional Chinese Medicine, Wenzhou Medical University, Wenzhou, China

**Keywords:** *Misgurnus anguillicaudatus*, *Lonicera japonica*, anti-inflammatory, lipopolysaccharide, microRNA

## Abstract

This study investigates the immunomodulatory effects of *Lonicera japonica* Thunb. extract, a perennial semi-evergreen vine of the Caprifoliaceae family, on lipopolysaccharide (LPS)-induced immune responses in loach (*Misgurnus anguillicaudatus*) through miRNA regulatory mechanisms. Small RNA libraries constructed from hepatic tissues of LPS-challenged (CK) and Lonicera japonica-treated (LJ) groups yielded 139.6 million clean reads with characteristic 21–23 nucleotide length distribution. Abundance analysis revealed significant differential expression patterns within the let-7 family: miR-let-7-9, miR-let-7-6, and miR-let-7–18 exhibited higher abundance in the infection group, whereas miR-let-7-1, miR-let-7-17, and miR-let-7–16 showed elevated abundance in the treatment group. Comparative profiling identified 55 differentially expressed miRNAs (41 upregulated, 14 downregulated), with animal-undef-351, animal-mir-21-6, and animal-undef-603 demonstrating the most significant expression differences (P<0.01). KEGG enrichment analysis highlighted predominant involvement in sphingolipid signaling pathway, glycerophospholipid metabolism, T cell receptor signaling pathway, and TNF signaling pathway. GO analysis revealed significant enrichment in biological processes related to glycosylation, cellular components of transcription regulator complexes, and molecular functions associated with double-stranded DNA binding. These findings demonstrate that *L. japonica* alleviates LPS-induced inflammation by modulating miRNA expression networks, particularly through sphingolipid metabolism and TNF signaling pathways, providing novel molecular evolutionary insights into the immunoregulatory mechanisms of traditional Chinese medicine.

## 1 Introduction

The loach (*Misgurnus anguillicaudatus*), a benthic fish species of the family Cobitidae, derives its scientific name from its ecological niche in lacustrine and pond sediments rich in humic substrates (1). This species is widely distributed across freshwater ecosystems in Asia, with primary habitats concentrated in the Yangtze River Basin of China, as well as in Korea and Japan (2–4). Recognized as a high-value aquaculture species, the loach is not only rich in high-quality proteins, vitamins, and essential amino acids (5), but also exhibits remarkable environmental adaptability, surviving under extreme conditions such as elevated temperatures (>30 °C), hypoxic environments, and intermittent drought (6, 7). Recent studies have revealed that bioactive components in loach tissues, including mucous polysaccharides and antioxidant enzymes, possess anti-inflammatory, hepatoprotective, and antitumor potential (8), driving its transition from traditional culinary use to medicinal applications. Owing to these attributes, the loach has emerged as a cornerstone species in China’s freshwater aquaculture industry (8). However, intensive farming practices are frequently challenged by pathogenic microbial infections (e.g., *Aeromonas hydrophila* and *Edwardsiella tarda*) and environmental stressors such as ammonia nitrogen accumulation, which trigger outbreaks of inflammatory diseases, thereby posing significant threats to aquaculture productivity and industry sustainability (9, 10).

Lipopolysaccharide (LPS), a major component of Gram-negative bacterial outer membranes, triggers systemic inflammation in fish by activating the Toll-like receptor 4 (TLR4)/NF-κB signaling pathway, leading to excessive production of pro-inflammatory cytokines such as TNF-α and IL-1β (11–14). Honeysuckle (*L. japonica*), a traditional Chinese herb, contains bioactive compounds including luteolin (LUT), chlorogenic acid, and caffeic acid, which exhibit broad-spectrum anti-inflammatory, immunomodulatory, and antioxidant properties (15–17). Previous studies have demonstrated that honeysuckle extracts alleviate mammalian inflammatory injuries via TLR4 pathway inhibition (18), yet their regulatory effects on LPS-induced inflammation in fish remain unexplored.

MicroRNAs (miRNAs), a class of endogenous non-coding RNAs (~22 nt in length), regulate gene expression by binding to the 3’ untranslated region (UTR) of target mRNAs, playing pivotal roles in immune responses and cellular homeostasis (19). Since the discovery of the first miRNA (lin-4) in Caenorhabditis elegans in 1993 (20), high-throughput sequencing technologies have identified hundreds of conserved miRNAs in model fish species such as zebrafish (*Danio rerio*) (21, 22). Recent advances further highlight the critical functions of miRNAs in stress adaptation and disease resistance of non-model aquaculture species (23, 24), providing novel insights into deciphering anti-pathogenic mechanisms.

Although existing studies have focused on loach immunity (8, 25, 26), the molecular mechanisms underlying herbal intervention in LPS-induced inflammatory responses are largely unknown. This study pioneers the integration of small RNA sequencing and molecular biology approaches to systematically analyze the miRNA expression profiles in loach muscle tissues following LPS challenge and honeysuckle treatment, which contributed to further understanding of the anti-inflammatory mechanism of Honeysuckle, and thus provided a basis for further optimization of artificial breeding methods of loach.

## 2 Materials and methods

### 2.1 Experimental animal and LPS treatment

A total of 300 healthy loaches of similar size were purchased from aquatic animal breeding base of Zhejiang Province and randomly divided into 6 groups with an average of 50 in each group. Normal feeding for 3 days, 20 g feed per day. On the fourth day, three groups were given Chinese herbal powder, that is, 20 g of feed and 10g of Chinese herbal medicine, and they were set as the experimental group, and the other three groups were given the same amount of normal feeding and were set as the control group. After 12 h, each loach in both the control and treatment groups was intraperitoneally injected with 100 μL of LPS solution (60 mg/100 μL, dissolved in phosphate-buffered saline (PBS)). The LPS used in this study was purchased from Beijing Solarbio Science & Technology Co., Ltd. The PBS solution preparation method is as follows: 8.00 g NaCl, 0.20 g KCl, 1.44 g Na_2_HPO_4_, and 0.24 g KH_2_PO4 and dissolved in 800 mL of distilled water. Adjust the HCl solution to pH 7.4, and supplementary distilled water to make a constant volume of 1 liter from then on. Two sets of samples were observed for 3,6,12 and 24 h. The hepatic tissue of the sample was ground and quickly frozen with liquid nitrogen and stored in the refrigerator at -80 °C.

### 2.2 Total RNA extraction, cDNA library preparation

Total RNA was extracted from loach tissues using Trizol Reagent (Invitrogen, Carlsbad, CA, USA) followed by DNase I (RNase-free) treatment to eliminate genomic DNA contamination. RNA purity and concentration were assessed using a NanoDrop 2000 spectrophotometer (Thermo Fisher Scientific, Wilmington, DE, USA), while RNA integrity was evaluated via an Agilent 2100 Bioanalyzer (Agilent Technologies, Santa Clara, CA, USA). Samples meeting the following criteria were selected for subsequent analysis: 28S/18S rRNA ratio ≥1.0, RNA Integrity Number (RIN) ≥7.0, OD260/280 = 1.8-2.2, and OD260/230 ≥2.0. To enrich small RNA molecules (18–30 nt), RNA fragments were size-selected using 15% denaturing polyacrylamide gel electrophoresis (PAGE). The purified small RNAs were ligated with 3′ and 5′ adapters using T4 RNA ligase (New England Biolabs, Ipswich, MA, USA). Reverse transcription was performed with adapter-specific primers, followed by PCR amplification to generate cDNA libraries. PCR products were purified via 3.5% agarose gel electrophoresis to isolate fragments of the desired size (140–160 bp), thereby removing unincorporated primers, primer dimers, and adapter contaminants. The final cDNA libraries were quantified by qPCR and subjected to paired-end sequencing (PE150) on an Illumina HiSeq platform (Shanghai Personal Biotechnology Co., Ltd., Shanghai, China).

The original sequence was sequenced by using the script independently developed by Shanghai Personal Biotechnology Co., Ltd. to remove the joint, and then mass cutting was performed according to the quality of the sequence. Specifically, the original sequence was searched with 5 base lengths as a window. When the average sequencing quality of bases in the window was lower than 20, the part starting from the front of the window was truncated and discarded. The filter sequences, namely clean reads, were obtained. The number of Clean Reads (Total Reads) whose sequence length was more than 18 nt and less than 36 nt was analyzed. The identical sequences in a single sample were de-processed and sequence abundance was counted to obtain Unique Reads for subsequent analysis.

### 2.3 Small RNA annotation

Using Blast (27), Unique Reads were compared with the Rfam13 database to screen four known classes of ncRNAs, including rRNA, tRNA, snRNA and snoRNA. The screening criteria are no more than 2 perfect matches or mismatches.

Reads that were not annotated to the above four types of ncRNAs were screened from Blast and all mature miRNA sequences in miRBase22 (http://www.mirbase.org/, Griffths -Jones S, 2004) for conserved miRNAs. The screening criteria are perfect match or mismatch number no more than 2.

we summarized the comparison and annotation of all small RNAs with other types of RNAs. Small RNA sequences are short, and it is generally easy to match different fragments, so there are multiple annotation results. In order to make each small RNA have a unique comment, the existing comment results are sorted according to the priority of known miRNA > rRNA > tRNA > snRNA > snoRNA > novel miRNA. At the same time, because different base positions of miRNAs may have different base preferences, base preference analysis was performed for known miRNAs.

### 2.4 Differentially expressed miRNAs analysis

In order to observe the conserved miRNA among species, miRNA of Loach was Blast compared with the mature miRNA sequences of related species in the miRBase database, and the Reads Count value of miRNA was calculated according to the number of conserved miRNA sequences. Then, the conservative miRNA expression data of the control group and the treatment group were collated, and DESeq (version 1.18.0, Anders S and Huber W, 2010) was used to analyze the miRNA with differential expression. They were mainly screened according to the expression multiple difference (|fold change| > 2) and the significance of expression difference (p-value < 0.05), and finally the results were obtained for statistics. At the same time, in order to judge the expression patterns of the obtained differentially expressed genes under different experimental conditions, we used R language Pheatmap software package to conduct bidirectional cluster analysis of genes and samples. The distance was calculated by Euclidean method and the Complete Linkage method was used for hierarchical clustering.

### 2.5 Target gene prediction and functional analysis of miRNAs

Because miRNAs bind to target sites mainly through complementary pairing. Therefore, we used miranda to predict miRNA target genes in animals and psRobot_tar to predict miRNA target genes in plants. Then, GO functional enrichment analysis was performed on the target genes of different miRNAs to obtain GO functional items that were significantly enriched in the target genes of different miRNAs, thus displaying relevant biological functions. (Map all genes to each Term in the Gene Ontology database, and calculate the number of target genes for each Term. Hypergeometric distribution was used to calculate the Term of significant enrichment of target genes against the background of the whole genome. The number of target genes contained in different levels of each KEGG Pathway was then counted to determine the metabolic pathways and signaling pathways that the target genes were mainly involved in (hypergeometric distribution was used to calculate the pathways with significant enrichment of target genes against the background of the whole genome).

### 2.6 Quantitative Real-Time PCR Validation

In order to verify the accuracy and reliability of the sequencing results, we selected qRT-PCR based on the principle of │log2(fc)│>1 and fpkm > 10. A total of 7 genes (5 down-regulated and 2 up-regulated) were selected for verification, and primers were designed by DNAstar (primer design is shown in **Table 1**). The qPCR was verified by SYBR^®^Green Supermix (TransGen Biotech) method with primer length of 18–24 bp as standard.

**Table 1:** Information of RT-qPCR primers.

miRNA	Forward primer 5’-3’
animal-undef-180	TGTAACAGCAACTCCATGTGGAA
animal-undef-459	AATCACTAACCTCACTACCAGG
animal-mir-122-2	TGGAGTGTGACAATGGTGTTTGT
animal-mir-181-4	CACATTCATTGCTGTCGGTGGGT
animal-mir-22-2	CGTTCTTCACTGGCTAGCTTTA
animal-mir-29-11	GCTGAATTCATATGGTGCCATAGA
animal-undef-334	TCGTGTCTTGTGTTGCAGCCAG

## 3 Results

### 3.1 Analysis of small RNA sequencing results

**Figure 1:**
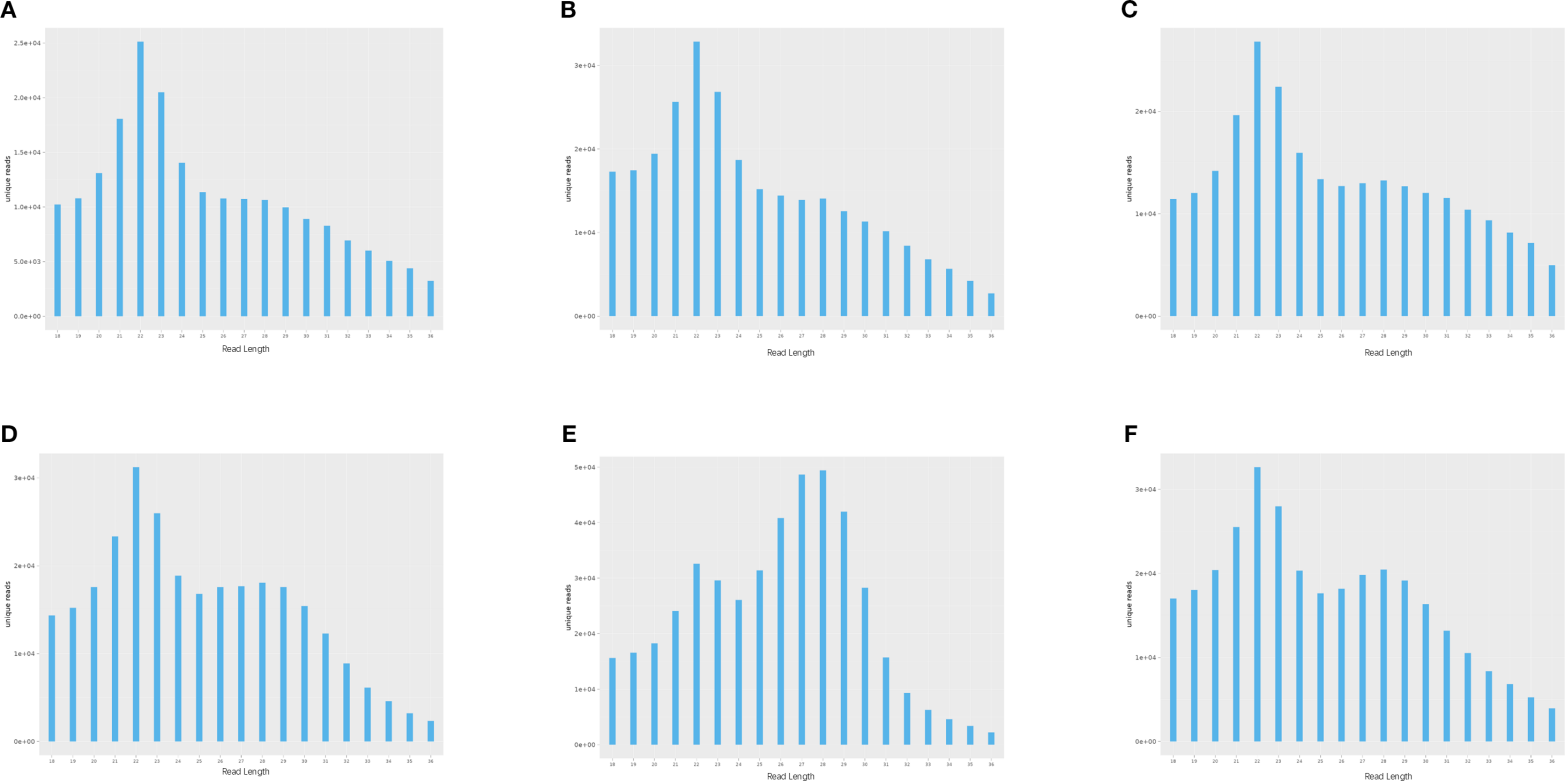
Length-specific distribution of deduplicated unique reads. (X: Read Length [nt]; Y: Abundance [×10^4^]): **(A)** CK1, **(B)** CK2, **(C)** CK3, **(D)** LJ1, **(E)** LJ2, **(F)** LJ3.

**Figure 2:**
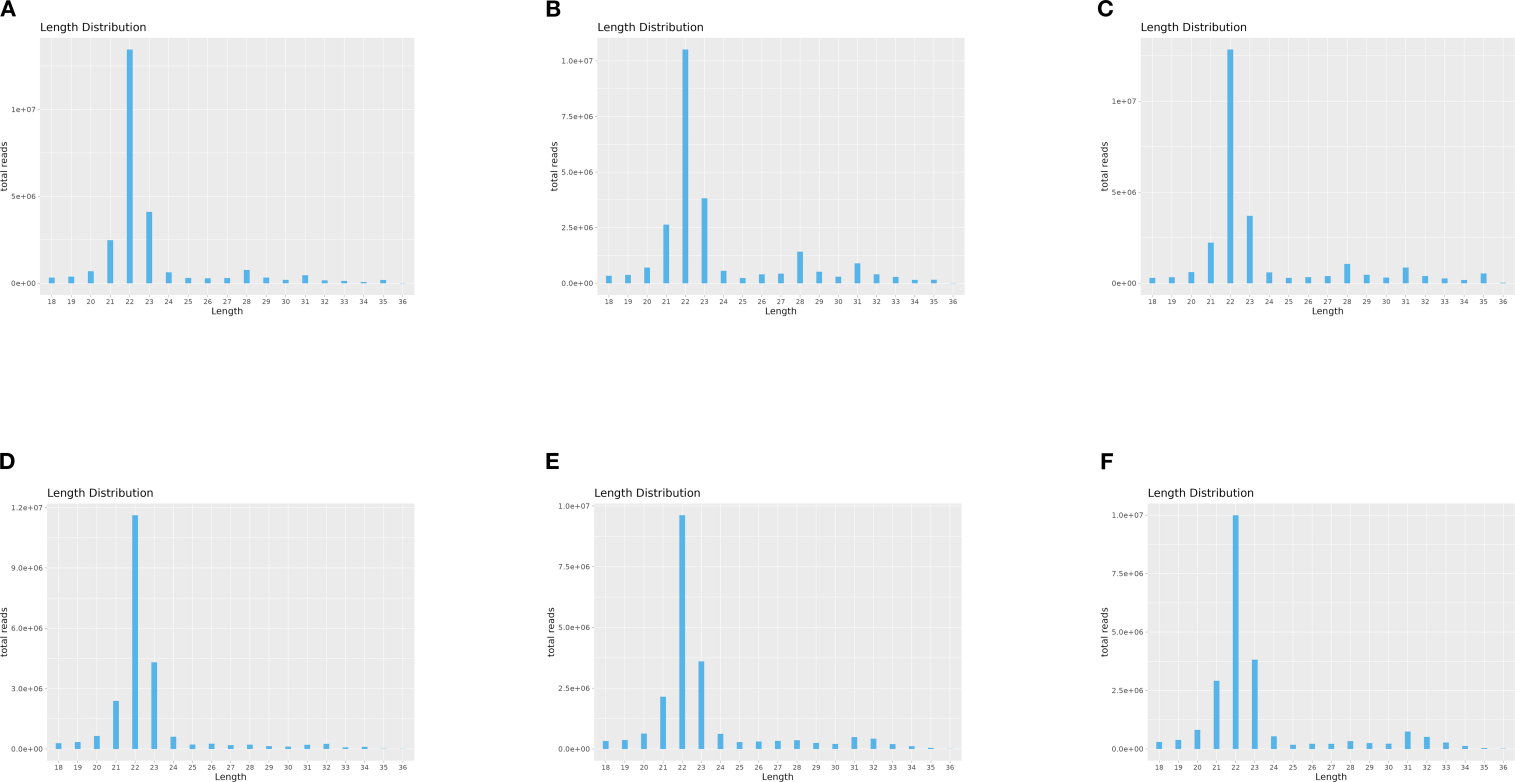
Length-specific distribution of deduplicated total reads. (X: Read Length [nt]; Y: Abundance [×10^4^]): **(A)** CK1, **(B)** CK2, **(C)** CK3, **(D)** LJ1, **(E)** LJ2, **(F)** LJ3.

To investigate the expression dynamics of miRNAs in loach (*M. anguillicaudatus*) under pathogenic infection and Honeysuckle (*L. japonica*) treatment, total RNA was extracted from hepatic tissues of both experimental (Honeysuckle-fed) and control groups, followed by the construction of six independent small RNA sequencing libraries. Deep sequencing generated 144.7 million raw reads across all libraries. After rigorous quality control procedures including adapter trimming, removal of contaminated reads, and elimination of low-quality sequences, 139.6 million high-quality clean reads were retained for subsequent analysis. Notably, the proportion of high-quality clean reads exceeded 95.6% in all six libraries (**Table 2**). Subsequently, we performed length distribution analysis on high-quality clean reads (total reads) ranging from 18 to 36 nucleotides (nt). Following deduplication to eliminate redundant sequences within individual samples, sequence abundance profiling revealed that small RNAs were predominantly enriched in the 21–23 nt range, with 22 nt sequences constituting the most abundant population (**Figure 1** and **Figure 2**). Notably, this size distribution pattern aligns with canonical characteristics of animal miRNAs, thereby substantiating the biological relevance of our sequencing data.

**Table 2:** Raw data statistics.

Sample	Raw reads	Clean reads	Clean reads%
CK1	26082271	25283783	96.939
CK2	25215222	24167742	95.846
CK3	26542407	25770796	97.093
LJ1	22731956	22039901	96.956
LJ2	21197521	20349467	95.999
LJ3	22932306	21945176	95.695

### 3.2 Identification of miRNAs in hepatic tissues of loach

**Figure 3:**
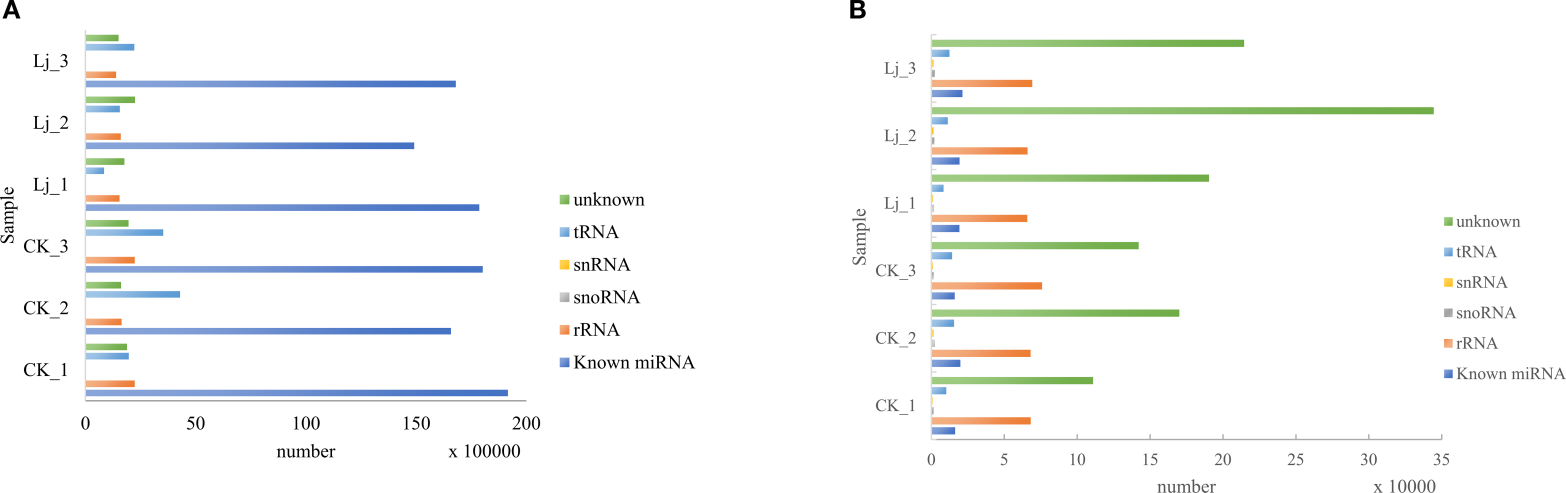
Small RNA Annotation: **(A)** Total Reads, **(B)** Deduplicated Reads.

The clean reads derived from both infected and control groups were systematically classified and annotated into three categories: known RNAs, unannotated RNAs, and non-coding RNAs (ncRNAs) including ribosomal RNA (rRNA), transfer RNA (tRNA), small nuclear RNA (snRNA), and small nucleolar RNA (snoRNA). Bioinformatic analysis revealed that the proportion of known RNAs in deduplicated clean reads ranged between 4% and 8% across groups (**Table 3**), whereas this proportion exceeded 68% prior to deduplication (**Table 4**). This striking reduction in known RNA abundance post-deduplication (from >68% to <10%) demonstrates significant dynamic changes in sequence complexity, suggesting effective removal of redundant transcriptional fragments during miRNA enrichment (**Figure 3**).

**Table 3:** Statistical results of miRNA in each sample (Unique).

Sample	Known miRNA	rRNA	snoRNA	snRNA	tRNA	Unknown miRNA
CK1	16298 (7.83%)	68155 (32.75%)	1533 (0.74%)	867 (0.42%)	10286 (4.94%)	110948 (53.32%)
CK2	19908 (7.17%)	68074 (24.52%)	2473 (0.89%)	1639 (0.59%)	15534 (5.59%)	170046 (61.24%)
CK3	16074 (6.40%)	75890 (30.22%)	1717 (0.68%)	1032 (0.41%)	14228 (5.67%)	142170 (56.62%)
Lj1	19294 (6.72%)	65758 (22.91%)	1932 (0.67%)	1224 (0.43%)	8398 (2.93%)	190436 (66.34%)
Lj2	19378 (4.36%)	66029 (14.84%)	2079 (0.47%)	1532 (0.34%)	11280 (2.54%)	344509 (77.45%)
Lj3	21376 (6.65%)	69222 (21.52%)	2424 (0.75%)	1679 (0.52%)	12445 (3.87%)	214523 (66.69%)

Column 1 (Sample ID) lists specimen identifiers; columns 2–7 indicate deduplicated read counts annotated to distinct RNA categories, including Known miRNA, rRNA, snoRNA, snRNA, tRNA, Unknown miRNA.

**Table 4:** Statistical results of miRNA in each sample (Total).

Sample	Known miRNA	rRNA	snoRNA	snRNA	tRNA	Unknown miRAN
CK1	19162329 (75.79%)	2237875 (8.85%)	20122 (0.08%)	11665 (0.05%)	1965785 (7.77%)	1886006 (7.46%)
CK2	16579908 (68.60%)	1640311 (6.79%)	26375 (0.11%)	16846 (0.07%)	4289971 (17.75%)	1614330 (6.68%)
CK3	18021510 (69.93%)	2232502 (8.66%)	21545 (0.08%)	11676 (0.05%)	3528303 (13.69%)	1955259 (7.59%)
Lj1	17863559 (81.05%)	1543619 (7.00%)	16856 (0.08%)	7160 (0.03%)	845113 (3.83%)	1763593 (8.00%)
Lj2	14911944 (83.28%)	1602422 (7.87%)	19378 (0.10%)	10372 (0.05%)	1557029 (7.65%)	2248321 (11.05%)
Lj3	16798062 (76.55%)	1389583 (6.33%)	21005 (0.10%)	13863 (0.06%)	2219461 (10.11%)	1503201 (6.85%)

Column 1 (Sample ID) lists specimen identifiers; columns 2–7 indicate deduplicated read counts annotated to distinct RNA categories, including Known miRNA, rRNA, snoRNA, snRNA, tRNA, Unknown miRNA.

**Figure 4:**
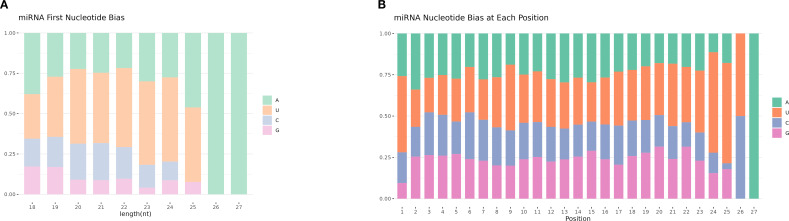
miRNA base preference. **(A)** The first base distribution of miRNAs of different lengths. The abscissa indicates sequences of different lengths; the ordinate indicates the percentage of the first base of each length of miRNA. **(B)** miRNA site base distribution.

Different base locations of miRNAs may have different base preferences. Therefore, base preference analysis of known miRNAs found that the 5 ‘end had a strong U tendency, that is, the typical miRNA base ratio caused by Dicer enzymes and DCL enzymes recognizing and cutting precursor miRNAs. Dicer and DCL enzymes recognize and cut the precursor miRNA, and its 5’ end has a strong U tendency. Through base preference analysis of miRNA, the typical miRNA base ratio was obtained (**Figure 4**).

### 3.3 Differentially expressed miRNAs identification

**Figure 5:**
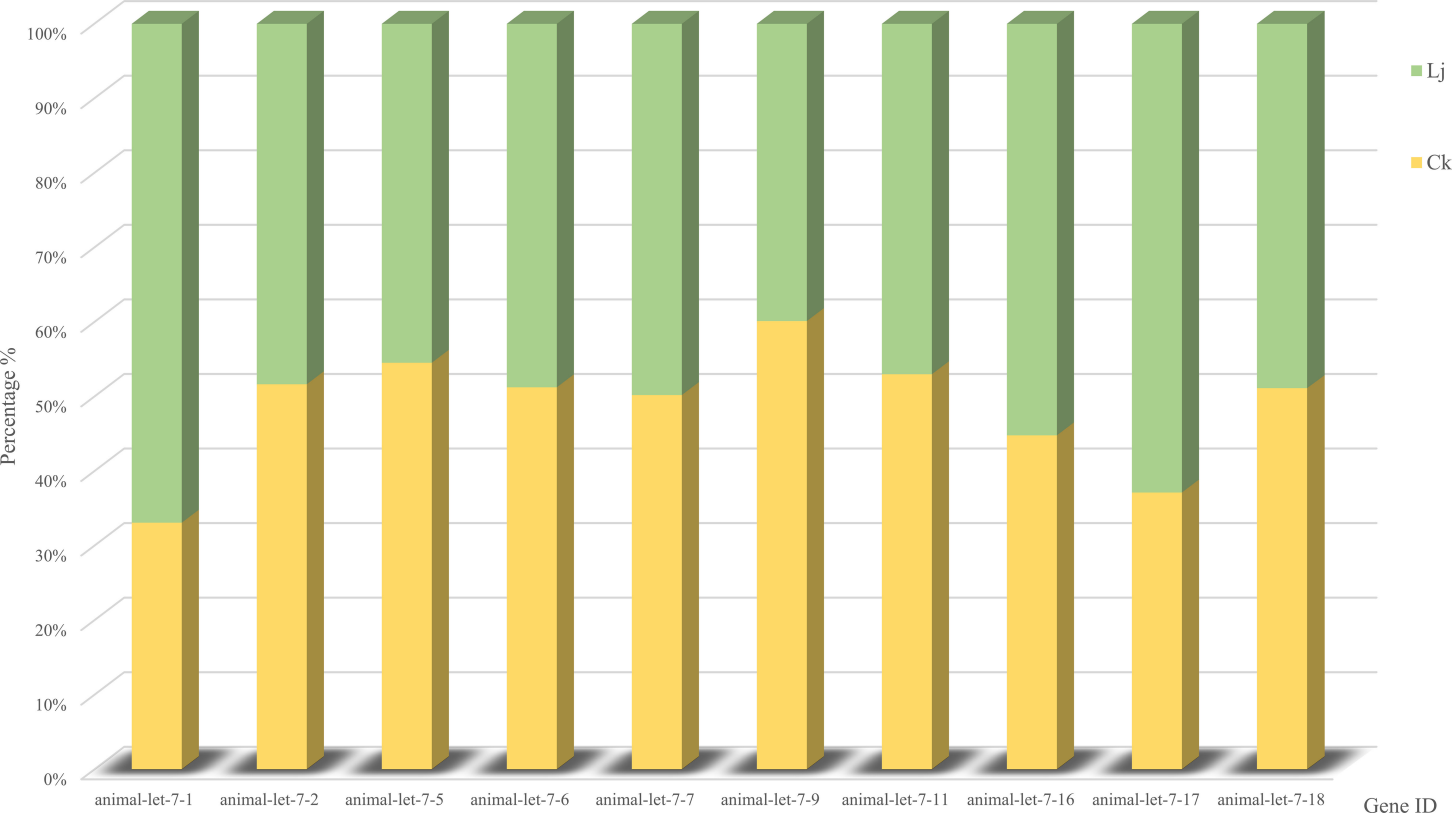
Comparative miRNA expression abundance profiles: infection group *vs*. control group.

Through alignment with evolutionarily conserved miRNAs, we quantified miRNA read counts across experimental groups (**Table 5**). Comparative analysis revealed that animal-let-7-9, animal-let-7-6, animal-let-7–18 exhibited statistically significant upregulation in the infected group compared to the honeysuckle-treated group. Conversely, animal-let-7-1, animal-let-7-17, and animal-let-7–16 demonstrated marked transcriptional activation following honeysuckle intervention, with fold-change values exceeding 2.5 (**Figure 5**). These differential expression patterns suggest pathogen-responsive and phytotherapeutic regulatory roles within the let-7 miRNA family.

**Table 5:** miRNA Expression table.

ID	CK1	CK2	CK3	Lj1	Lj2	Lj3
animal-let-7-1	19	15	12	14	19	60
animal-let-7-2	320	150	308	237	182	309
animal-let-7-3	0	0	0	0	1	0
animal-let-7-4	0	0	0	0	0	0
animal-let-7-5	29	4	15	5	20	15
animal-let-7-6	4220	3778	3451	2643	2201	6045
animal-let-7-7	28	49	40	27	31	58
animal-let-7-8	0	0	0	1	2	1
animal-let-7-9	168	152	128	139	74	84
animal-let-7-10	0	0	0	1	0	0
animal-let-7-11	151	195	192	96	127	254
animal-let-7-12	1	0	0	0	0	0
animal-let-7-13	0	11	0	11	0	3
animal-let-7-14	8	0	0	0	0	0
animal-let-7-15	0	0	1	0	0	0
animal-let-7-16	13	25	44	15	40	46
animal-let-7-17	410	337	345	311	322	1215
animal-let-7-18	1054	1559	906	920	930	1512
animal-let-7-19	0	0	1	0	0	0

**Figure 6:**
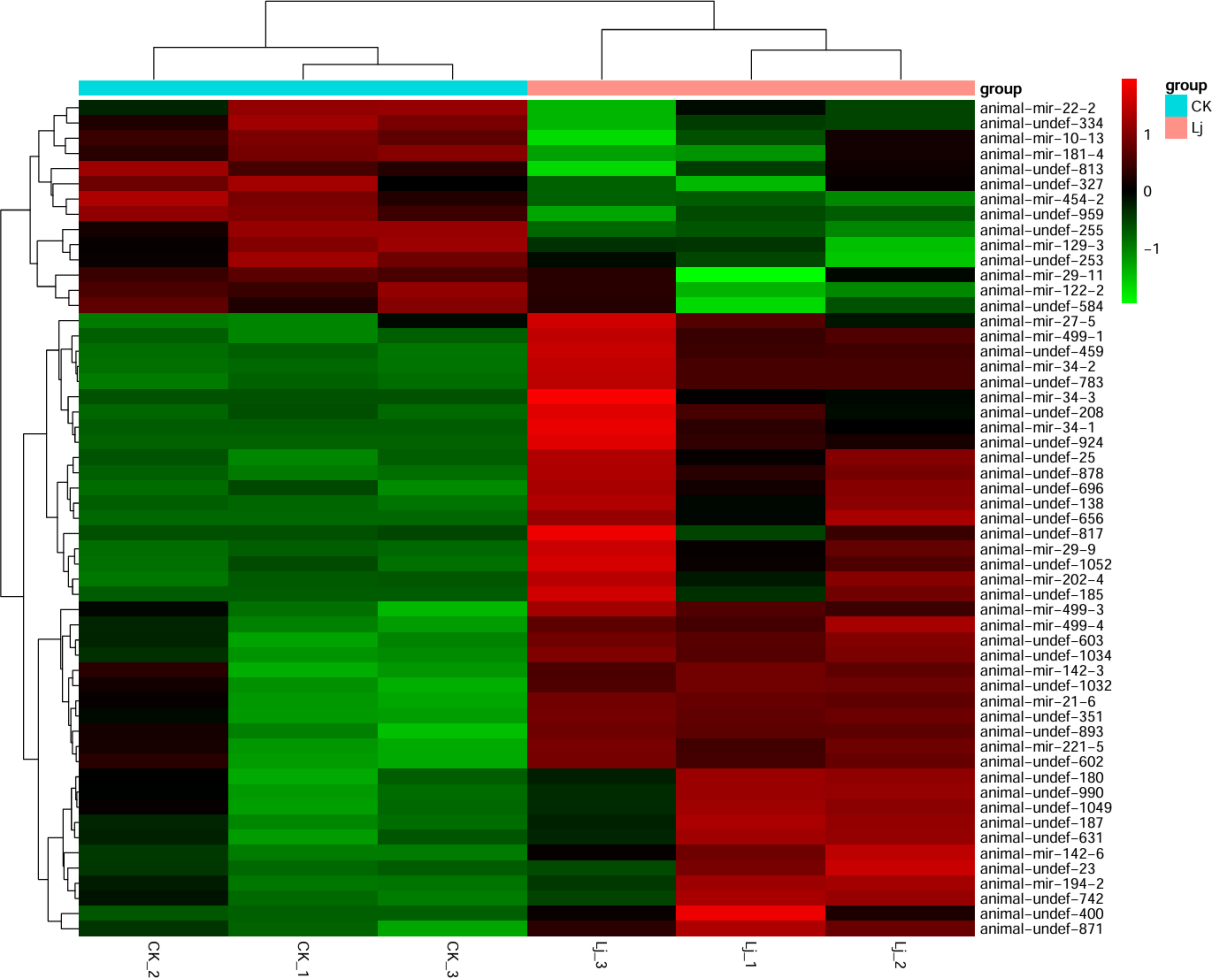
Cluster map of differentially expressed miRNAs. Red color indicates elevated expression and green color indicates decreased expression.

Comparing the expression differences of miRNA between LJ group and CK group, 55 differentially expressed genes were found (**Figure 6**), of which 41 were significantly up-regulated and 14 were significantly down-regulated. The expression profiles of all miRNA in group LJ and group CK are shown in the heatmap in **Figure 5**. Notably, among these differentially expressed miRNAs, animal-undef-351, animal-mir-21-6, and animal-undef-603 exhibited the most pronounced expression differences (P<0.01). Also, we found animal-mir-22-2, animal-mir-181-4, animal-mir-122-2, animal-mir-454-2, animal-mir-10-13, animal-mir-29-11, animal-mir-129–3 Down-regulated trends were observed in all miRNAs, suggesting that the transcription levels of these miRNAs may be regulated by common factors in LJ (**Table 6**).

**Table 6:** Details of the DE miRNA.

Id	Basemean	Foldchange	Log2foldchange	Pval	Regulation
animal-undef-1049	11782.96475	9.188399756	3.199813625	0.030968759	Up Regulation
animal-mir-22-2	10932.25366	0.274372355	-1.865792969	0.028245309	Down Regulation
animal-undef-631	8359.75076	12.82052138	3.680383029	0.013238321	Up Regulation
animal-mir-34-2	7827.016943	393.8993548	8.621683243	0.01456995	Up Regulation
animal-undef-783	6766.428457	438.654428	8.776941022	0.013794361	Up Regulation
animal-undef-584	3902.373138	0.256541954	-1.962733316	0.013675702	Down Regulation
animal-undef-180	3138.706219	6.671169252	2.737939644	0.031297186	Up Regulation
animal-undef-138	2963.995844	17.36082078	4.117763252	0.002987859	Up Regulation
animal-mir-181-4	2522.927278	0.330895191	-1.595553772	0.014639291	Down Regulation
animal-undef-871	2409.348114	3.123950705	1.643371688	0.014347344	Up Regulation
animal-mir-202-4	1536.967306	10.51788194	3.394772303	0.008876062	Up Regulation
animal-mir-21-6	1444.895181	8.065595221	3.011781005	2.77E-05	Up Regulation
animal-undef-602	1395.845333	3.749883657	1.906845836	0.0068776	Up Regulation
animal-mir-122-2	1136.901073	0.219093102	-2.190384033	0.015019224	Down Regulation
animal-undef-459	1125.430295	395.3382881	8.626943875	0.010823413	Up Regulation
animal-undef-25	884.6417133	19.17850255	4.261418174	0.002346502	Up Regulation
animal-mir-454-2	818.333124	0.38667082	-1.370822198	0.033976037	Down Regulation
animal-undef-1034	764.4187056	4.76921588	2.253752088	0.000955974	Up Regulation
animal-undef-334	735.7824428	0.300445811	-1.73482329	0.009225079	Down Regulation
animal-undef-603	734.3402938	5.901694925	2.561129346	0.000238207	Up Regulation
animal-undef-255	720.6407293	0.15042995	-2.732836262	0.001699442	Down Regulation
animal-undef-813	699.6699154	0.401108961	-1.317933899	0.034520118	Down Regulation
animal-undef-1052	683.6238279	2.948699038	1.56007858	0.025175416	Up Regulation
animal-undef-696	679.4901727	2.628131942	1.394037706	0.029581107	Up Regulation
animal-undef-351	602.0050031	8.888342593	3.151914425	1.68E-05	Up Regulation
animal-mir-499-1	582.6709591	3.153393337	1.656905135	0.009348807	Up Regulation
animal-mir-499-3	569.4320395	2.621984405	1.390659105	0.039496677	Up Regulation
animal-undef-1032	510.1540078	4.284071314	2.098982496	0.003925448	Up Regulation
animal-mir-221-5	491.9805404	2.669765069	1.416712795	0.044312765	Up Regulation
animal-mir-29-11	484.8379197	0.264120426	-1.920732215	0.006909173	Down Regulation
animal-undef-742	406.3747122	9.383100689	3.230064747	0.029093662	Up Regulation
animal-undef-187	392.9791336	32.07146034	5.003218142	0.00579882	Up Regulation
animal-mir-142-3	338.6001148	3.642439439	1.864904985	0.01278489	Up Regulation
animal-mir-194-2	255.8252466	11.29848752	3.498057753	0.016862136	Up Regulation
animal-mir-10-13	195.2983477	0.362554127	-1.463731698	0.035091219	Down Regulation
animal-undef-878	182.9775029	2.827508206	1.499531211	0.029376013	Up Regulation
animal-mir-129-3	159.6342067	0.173248604	-2.529084365	0.007749348	Down Regulation
animal-undef-893	156.0708447	3.241193619	1.696525205	0.029246391	Up Regulation
animal-undef-185	116.4199725	Inf	Inf	0.000493451	Up Regulation
animal-undef-990	112.924222	6.264910706	2.647293947	0.033714318	Up Regulation
animal-mir-34-3	110.1466648	Inf	Inf	0.01868192	Up Regulation
animal-undef-327	103.3963438	0.205227457	-2.284704337	0.002652137	Down Regulation
animal-mir-499-4	95.2790711	3.095362026	1.630108153	0.04170206	Up Regulation
animal-undef-253	73.24061181	0.304316387	-1.716356073	0.043706763	Down Regulation
animal-mir-29-9	62.42991517	3.452772283	1.787755189	0.025949776	Up Regulation
animal-mir-34-1	49.15137545	Inf	Inf	0.004380187	Up Regulation
animal-undef-208	29.12362958	3.919111417	1.970526588	0.045623412	Up Regulation
animal-undef-959	22.88808307	0.203187642	-2.299115435	0.036554846	Down Regulation
animal-mir-27-5	22.59189593	4.642723711	2.214971429	0.032208271	Up Regulation
animal-undef-924	21.65121781	Inf	Inf	0.000308585	Up Regulation
animal-undef-400	20.02336313	135.9554882	7.086990581	0.016559208	Up Regulation
animal-mir-142-6	15.5809598	14.22434384	3.830290199	0.011757006	Up Regulation
animal-undef-23	10.38216178	12.17799069	3.60620421	0.042048201	Up Regulation
animal-undef-817	8.096682713	38.87977687	5.280948034	0.034441586	Up Regulation
animal-undef-656	5.684530263	Inf	Inf	0.022044717	Up Regulation

### 3.4 Enrichment analysis of GO and KEGG pathways of target genes predicted by miRNA

**Figure 7:**
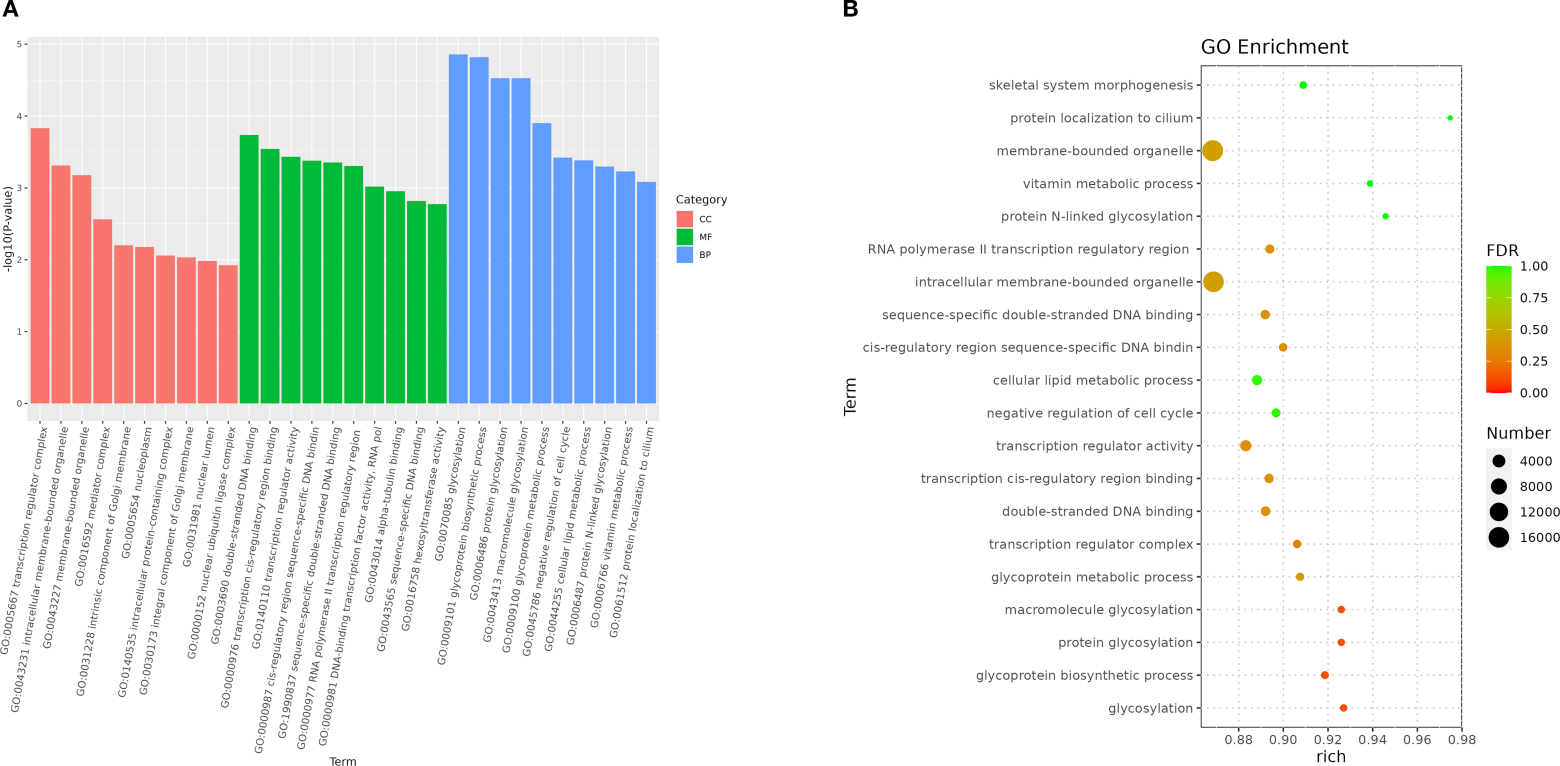
Enriched GO of genes targeted by DEM in 2 groups. **(A)** GO enrichment bar plot of differentially expressed miRNA target genes. **(B)** Bubble Plot of GO Enrichment Analysis for Target Genes of Differentially Expressed miRNAs.

In this experiment, a total of 91,596 target genes were predicted, among which 150 miRNAs had target genes. At the same time, a target gene might correspond to multiple targets, so a total of 913,690 target sites were predicted. Then in the GO enrichment, we selected the top 10 biological process (BP), cellular component (CC), and molecular function (MF) directories. In the category of biological processes, most of the noted targets involve glycosylation and Glycoprotein biosynthetic processes. Within the cell component category, most targets were assigned to transcription regulator complex and intracellular membrane-bounded organelle. In the molecular function category, most targets are associated with double-stranded DNA binding and transcription cis-regulatory region binding (**Figure 7**).

**Figure 8:**
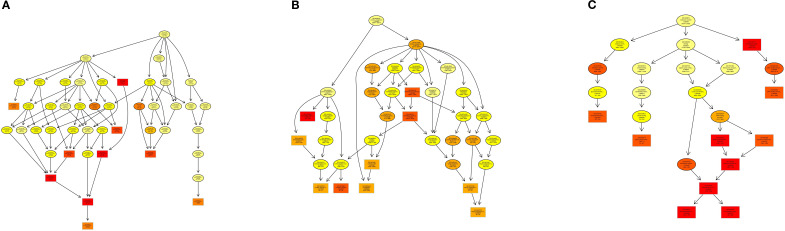
DAG visualization of GO enrichment analysis: **(A)** Biological Process (BP), **(B)** Cellular Component (CC), **(C)** Molecular Function (MF).

The Gene Ontology (GO) enrichment analysis revealed distinct hierarchical patterns across functional categories: Biological Process (BP): A functionally coherent hierarchy spanning from ubiquitin-dependent endocytosis (GO:0048497) through macromolecule modification (GO:0043412) to protein glycosylation (GO:0006486) exhibited statistically significant enrichment, indicating systematic activation of post-translational modification pathways (**Figure 8A**). Cellular Component (CC): The parent-child relationship between membrane-bounded organelle (GO:0043227) and its descendant term intracellular membrane-bounded organelle (GO:0043231) showed pronounced enrichment, highlighting compartment-specific regulatory mechanisms (**Figure 8B**). Molecular Function (MF): Enriched GO terms within the DNA binding (GO:0003677) and extracellular vesicle biogenesis (GO:0140115), suggesting coordinated regulation of genomic integrity maintenance and intercellular communication processes (**Figure 8C**).

**Figure 9:**
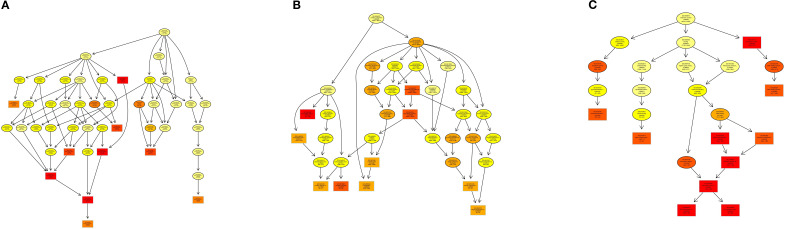
Enriched KEGG of genes targeted by DEM in 2 groups. **(A)** KEGG enrichment bar plot of differentially expressed miRNA target genes. **(B)** Bubble Plot of KEGG Enrichment Analysis for Target Genes of Differentially Expressed miRNAs.

**Figure 10:**
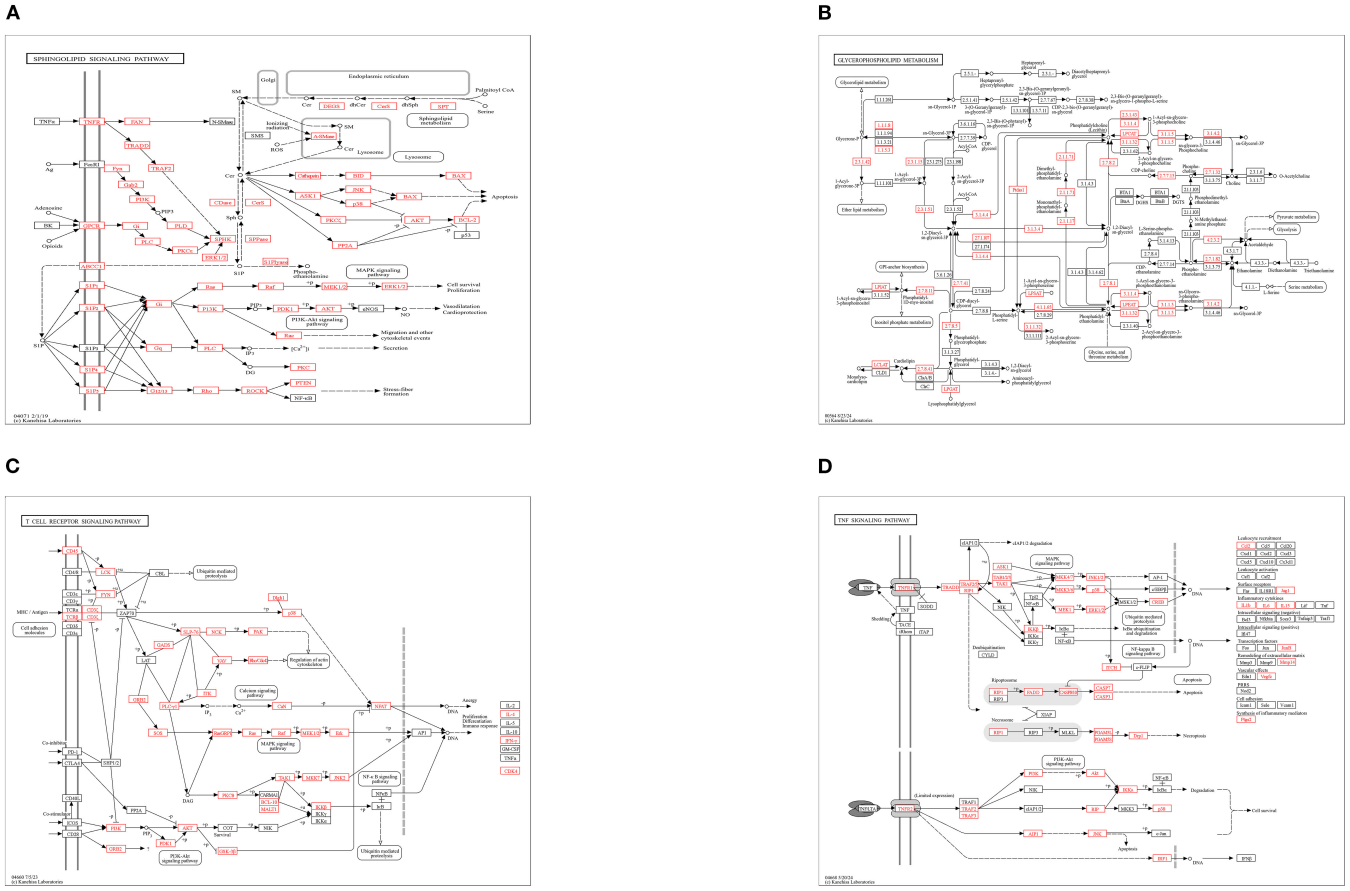
KEGG pathway enrichment analysis: **(A)** Sphingolipid Signaling Pathway, **(B)** Glycerophospholipid Metabolism, **(C)** T-cell Receptor (TCR) Signaling Pathway, **(D)** TNF Signaling Pathway.

In addition, In the KEGG rich concentration (**Figure 9** and **Figure 10**), we found sphingolipid signaling pathway and glycerophospholipid metabolism involved in cell recognition and signal transduction, and T cell receptor involved in anti-inflammatory signaling pathway and TNF signaling pathway.

### 3.5 qRT-PCR consistency for selected miRNAs

**Figure 11:**
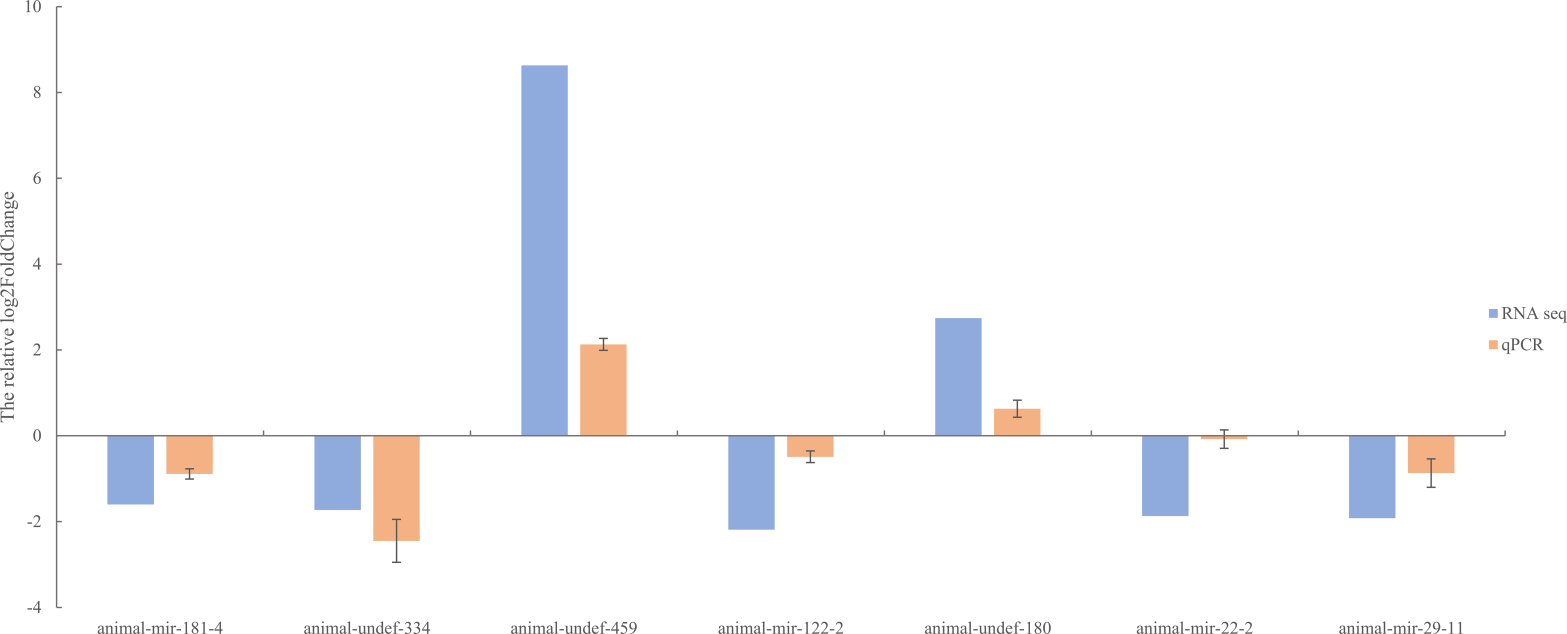
QPCR validation figure of differentially expressed miRNAs.

To further verify the differentially expressed miRNAs identified by RNA-seq, we used qRT-PCR for confirmation. Seven miRNAs (animal-undef-180, animal-undef-459, animal-mir-122-2, animal-mir-181-4, animal-mir-22-2, animal-mir-29-11, and animal-u) were selected ndef-334 performed qRT-PCR, and it was found that 2 up-regulated genes and 5 down-regulated genes showed the same regulatory trend with qRT-PCR compared with the control group. This indicated that the expression pattern of differentially expressed miRNA was consistent with the results of real-time PCR detection (**Figure 11**).

## 4 Discussion

The loach, a commercially vital freshwater aquaculture species in China, faces critical challenges in sustainable production due to inflammation-related diseases. Enhancing its growth performance and disease resistance remains a priority for aquaculture optimization. Honeysuckle (*L. japonica*), a traditional Chinese herb, contains bioactive compounds (e.g., chlorogenic acid and luteoloside) with demonstrated anti-inflammatory, immunomodulatory, and antimicrobial properties (28–30). Previous studies in lipopolysaccharide (LPS)-induced acute lung injury (ALI) murine models revealed that honeysuckle extracts significantly suppressed pro-inflammatory cytokines (TNF-α, IL-6) via modulation of the NF-κB pathway, concurrently improving survival rates (31). Nevertheless, the molecular mechanisms underlying herbal immunoregulation in aquatic species remain underexplored. Therefore, this study employed an integrated experimental design combining lipopolysaccharide (LPS) challenge with *L. japonica*-supplemented dietary intervention in loach, to systematically characterize miRNA expression dynamics.

In this study, a total of 20,349,467-25,283,783 clean reads were identified by sequencing technology. with clean read ratios exceeding 95%, confirming robust data integrity that meets established sequencing quality benchmarks for subsequent bioinformatic analyses. The six groups of clean reads were distributed in the range of 18–30 bp in length, mainly 21–23 bp, and clustered most at 22 bp. This is consistent with the typical sequence length distribution of Dicer derivatives.

Integrated analysis of small RNA sequencing data from *Aeromonas hydrophila*-infected and honeysuckle-treated loach groups identified 55 differentially expressed miRNAs (DEMs), comprising 41 upregulated and 14 downregulated species. Notably, several DEMs have been previously implicated in inflammatory regulation and muscle development: miR-21, miR-29, mir-142 and miR-122 were differentially modulated. Specifically, miR-21 was shown to preserve intestinal tight junction (TJ) barrier integrity via ROCK1-dependent upregulation of occludin expression, with its knockout exacerbating barrier dysfunction and inflammatory cascades (32–34). In *A. hydrophila*-infected grass carp, miR-142 exerted anti-apoptotic and anti-inflammatory effects by directly targeting TNFAIP-2 and GLUT-3 (35, 36). While miR-122 attenuated *A. hydrophila*-induced inflammation via suppression of IL-6, IL-15, and IL-1β (37), it concurrently disrupted TJ barrier proteins (occludin, claudin-2, claudin-5) and EGFR signaling, potentiating gastrointestinal hyperpermeability and secondary inflammation (38–42). Significant downregulation of miR-29 in stenotic mucosal regions of Castleman’s disease patients suggests its role in suppressing pathological hyperplasia-driven inflammation (43, 44). These findings collectively suggest that honeysuckle intervention may alleviate inflammatory phenotypes and enhance muscle quality in loach by orchestrating DEM expression networks (e.g., miR-21/miR-142) to suppress pro-inflammatory cascades while optimizing myogenic pathways.

KEGG pathway enrichment analysis revealed that target genes of differentially expressed miRNAs (DEMs) were significantly enriched in four pivotal signaling pathways: sphingolipid signaling, glycerophospholipid metabolism, T-cell receptor (TCR) signaling, and TNF signaling. As central regulators of lipid metabolism, sphingolipid and glycerophospholipid pathways orchestrate cellular proliferation, apoptosis, stress responses, and inflammatory cascades through bioactive lipid mediators such as sphingosine-1-phosphate (S1P) and lysophosphatidic acid (LPA) (45, 46). Pharmacological evidence demonstrates that gastrodin and gallic acid ameliorate sphingolipid dysregulation by upregulating S1P receptor 1 (S1PR1) expression, consequently suppressing pro-inflammatory cytokines (e.g., IL-1β and TNF-α) (47). Concurrently, glycerophospholipid metabolic perturbations elevate systemic inflammatory markers (e.g., CRP, IL-6), correlating with metabolic disorder progression (48). The TCR pathway reinforces immune tolerance via IL-10 secretion from regulatory T cells (Tregs), effectively curbing excessive inflammation (49), while TNF-α, a key effector of the TNF pathway, drives inflammatory cascades through NF-κB and MAPK activation. Notably, herbal interventions attenuate these pathways to mitigate inflammatory damage (50, 51). GO enrichment analysis revealed that the majority of differentially expressed genes were significantly enriched in the biological process of glycosylation, the cellular component of transcription regulator complexes, and the molecular function of double-stranded DNA binding-all of which are critically involved in inflammatory regulation. We propose that glycosylation modifications regulate inflammatory pathways (e.g., NF-κB) by modulating transcription factor stability and facilitating transcriptional condensate formation. Furthermore, the functional activity of double-stranded DNA-binding proteins is influenced by their glycosylation status, while the assembly of transcriptional condensates depends on DNA scaffolding. These three mechanisms synergistically orchestrate inflammatory resolution through coordinated regulation of gene expression, chromatin remodeling, and signal transduction cascades (52–58). Collectively, integrated KEGG and GO evidence suggests that honeysuckle dietary intervention exerts synergistic anti-inflammatory effects in loach by multi-target modulation of lipid metabolic reprogramming, immune tolerance establishment, and epigenetic remodeling.

## 5 Conclusion

In this study, based on miRNA transcriptome sequencing technology, the mechanism of LPS-mediated effects of honeysuckle on the immune function of Loach was investigated. The results showed that honeysuckle had certain effects on the immune function of loach. This study provides a new idea for the rational utilization of honeysuckle, and provides theoretical guidance for the healthy feeding of loach.

## Data Availability

The datasets presented in this study can be found in online repositories. The names of the repository/repositories and accession number(s) can be found below: https://www.ncbi.nlm.nih.gov/genbank/, SRX25348514.
